# Middle East Respiratory Syndrome Coronavirus Antibodies in Dromedary Camels, Bangladesh, 2015

**DOI:** 10.3201/eid2405.171192

**Published:** 2018-05

**Authors:** Ariful Islam, Jonathan H. Epstein, Melinda K. Rostal, Shariful Islam, Mohammed Ziaur Rahman, Mohammed Enayet Hossain, Mohammed Salim Uzzaman, Vincent J. Munster, Malik Peiris, Meerjady Sabrina Flora, Mahmudur Rahman, Peter Daszak

**Affiliations:** EcoHealth Alliance, New York, New York, USA (A. Islam, J.H. Epstein, M.K. Rostal, S. Islam, P. Daszak);; Institute of Epidemiology, Disease Control and Research, Dhaka, Bangladesh (S. Islam, M.S. Uzzaman, M.S. Flora, M. Rahman);; icddr,b, Dhaka (M.Z. Rahman, M.E. Hossain);; National Institutes of Health, Hamilton, Montana, USA (V.J. Munster);; University of Hong Kong School of Public Health, Hong Kong, China (M. Peiris)

**Keywords:** dromedary, camel, coronavirus, viruses, livestock trade, Bangladesh, serology, Middle East respiratory syndrome coronavirus, MERS-CoV

## Abstract

Dromedary camels are bred domestically and imported into Bangladesh. In 2015, of 55 camels tested for Middle East respiratory syndrome coronavirus in Dhaka, 17 (31%) were seropositive, including 1 bred locally. None were PCR positive. The potential for infected camels in urban markets could have public health implications and warrants further investigation.

Middle East respiratory syndrome coronavirus (MERS-CoV), discovered in 2012, can cause fatal respiratory disease in humans. Although MERS-CoV might have originated in bats, dromedary camels (*Camelus dromedarius*) are a natural host and likely source of human MERS-CoV infection (*1*,*2*). Camel trade is a major driver of MERS-CoV movement between Africa and the Arabian Peninsula (*3*), where most human cases have occurred. Rajastan, India, is a large breeding center for dromedaries, some of which are exported to Pakistan and Bangladesh. Seropositive dromedaries have been identified in Pakistan, but little is known about MERS-CoV in other parts of South Asia (*4*). In Bangladesh, camels are bred on farms and imported from India for sale in seasonal markets for ritual slaughter during religious festivals. Imported camels go directly to urban markets to be sold by traders and are a separate enterprise from farmed camels.

During the September–October 2015 festival of Eid-ul-Adha, we collected and tested for coronaviruses the specimens of 36 dromedary camels at an urban farm and 19 camels and 18 fat-tailed sheep at an urban market in the capital city of Dhaka (Table; Technical Appendix). The testing was conducted as part of the US Agency for International Development’s PREDICT program, which conducts surveillance in humans and animals for novel and select known zoonotic viruses, including MERS-CoV. We obtained information for each camel’s origin and age from market registries or breeders’ records. We also assessed and recorded the sex and apparent health status of each camel at specimen collection, at which time we collected blood, 2 nasal swab specimens, and 2 rectal swab specimens from each animal. We placed 1 set of each swab in lysis buffer (Nuclisens; bioMérieux, Marcy-l’Étoile, France) and 1 in viral transport medium. We separated and froze serum samples. We extracted total nucleic acid by using EasyMag (bioMérieux) and performed cDNA synthesis by using SuperScript III first-strand synthesis supermix (Invitrogen, Carlsbad, CA, USA) according to the manufacturer’s instructions. We performed pancoronavirus PCR targeting the *RdRp* gene (*5*) and MERS-CoV real-time PCR targeting the upstream envelope protein gene and nucleocapsid protein genes N2 and N3 (*6*). We screened serum samples by using a MERS-CoV ELISA (*7*) and confirmed the results by using a MERS-CoV pseudoparticle neutralization test (*8*).

**Table:** Selected characteristics and MERS-CoV laboratory results for dromedary camels, Bangladesh, 2015*

Characteristic	No. (%) camels		No. (%) positive for MERS-CoV, by laboratory test					
			rPCR (%)	ELISA (%, 95% CI)			ppNT (%, 95% CI)	
All camels								
Location								
Farm	36 (56)		0	6 (17, 6–33)			5 (14, 5–29)	
Market	19 (35)		0	11 (58, 34–80)			12 (63, 38–84)	
Origin								
Imported	31 (56)		0	16 (52, 33–70)			16 (52, 33–70)	
Bangladesh	24 (44)		0	1 (4, 1–21)			1 (4, 0–21)	
Sex								
M	29 (53)		0	6 (21, 8–40)			7 (24, 10–44)	
F	26 (47)		0	11 (42, 23–63)			10 (38, 20–59)	
Age group								
Adult, >2 y	44 (80)		0	16 (36, 22–52)			16 (36, 22–52)	
Juvenile, <2 y	11 (20)		0	1 (9, 1–41)			1 (9, 0–41)	
Body condition								
Poor	19 (35)		0	11 (58, 34–80)			12 (63, 38–84)	
Fair	6 (11)		0	1 (16, 1–64)			0	
Good	30 (55)		0	5 (17, 1–35)			5 (17, 06–35)	
Upper respiratory signs								
Yes	2 (4)		0	1 (50, 1–99)			0	
No	53 (96)		0	16 (30, 18–44			17 (32, 20–46)	
Farm camels								
Age group								
Adult, >2 y	8 (22)		0	0			0	
Juvenile, <2 y	28 (78)		0	6 (21, 8–41)			5 (18, 6–37)	
Sex								
M	18 (50)		0	1 (6, 1–27)			4 (22, 6–48)	
F	18 (50)		0	5 (28, 10–53)			1 (6, 1–27)	
Origin								
Bangladesh	24 (67)		0	1 (4, 1–21)			1 (4, 1–21)	
Imported	12 (33)		0	5 (42, 15–72)			4 (33, 9–65)	
Market camels								
Age group								
Adult, >2 y	3 (16)	0			1 (33, 1–90)		1 (33, 1–90)	
Juvenile, <2 y	16 (84)	0			10 (63, 35–85)		11 (69, 41–89)	
Sex								
M	11 (58)	0			6 (75, 23–83)		6 (55, 23–83)	
F	8 (42)	0			5 (45, 24–91)		6 (75, 35–97)	
Origin								
Bangladesh	0	0			0		0	
Imported	19 (100)	0			11 (58, 34–80)		12 (63, 38–84)	

*MERS-CoV, Middle East respiratory syndrome coronavirus; ppNT, pseudoparticle neutralization test; rPCR, real-time PCR.

Of the 36 camels on the farm, 24 were born there. The remaining 12 and all 19 market camels were imported from India (Table). All specimens tested negative for coronaviruses, including MERS-CoV, by PCR. ELISA showed 98.6% specificity and sensitivity compared with the pseudoparticle neutralization test. We detected MERS-CoV antibodies in 31% (95% CI 19%–45%) of camels; adults had a higher seroprevalence (36% [95% CI 22%–52%]) than juveniles (9% [95% CI 0.2%–41%]). Imported camels had a significantly higher seroprevalence (52% [95% CI 33%–70%]) than domestically bred camels (4% [95% CI 0.1%–21%]). Among the 5 seropositive farm camels, 1 was a domestically bred adult, whereas the other 4 were adults from India. Camels in the market had a higher seroprevalence (63% [95% CI 38%–85%]) than those on the farm (14% [95% CI 5%–30%]). All sheep serum samples were negative for MERS-CoV antibodies.

The findings of a higher MERS-CoV seroprevalence in adult camels (*9*) and the seronegativity in sheep are consistent with other studies (*8*). Only adult camels were found in the market. The finding of an adult seropositive camel, born on the farm, suggests that it was infected locally. No records indicate intermingling between farmed camels and those in markets. The finding of only 1 seropositive camel originating in Bangladesh suggests that if infection or exposure occurred on the farm, either viral circulation was limited or other seropositive camels had since been sold or removed. Juveniles are more likely to be actively infected than adults, and the limited juvenile sample size might explain our lack of virus detection among them (*9*).

Our findings suggest transmission of MERS-CoV has occurred among camels in Bangladesh, extending the previously reported range of this virus (up to ≈1,900 km east of Pakistan). Exactly where or when imported camels became infected is unclear. To date, no human cases of MERS-CoV have been reported in South Asia. The possibility of having MERS-CoV–infected camels in Dhaka, a populous city with ≈18 million persons, presents a potential risk for human outbreaks. Insufficient surveillance, behavioral differences in human–camel interactions compared with Middle Eastern societies, or differences in virus strains or human susceptibility might explain the lack of observed cases. Improved surveillance of camels along camel trade routes, camel herds in Dhaka, and persons who have close contact with camels will help assess the transboundary movement and the risk for zoonotic transmission in Bangladesh. Given the ubiquity of MERS-CoV in dromedary camels, the predictable seasonal movement of camels into Dhaka, and a higher incidence of infection in persons with frequent contact with camels (*10*), targeted public health messaging that promotes handwashing after contact with camels and avoidance of exposure to camel excreta might help reduce the risk for zoonotic MERS-CoV transmission.

Technical Appendix Sampling locations of dromedary camels, Bangladesh, 2015.
