# Supplementary material for: Middle East Respiratory Syndrome Coronavirus Antibodies in Dromedary Camels, Bangladesh, 2015
Source: Emerg Infect Dis. 2018 May;24(5):926–8. doi: 10.3201/eid2405.171192 (PMC5938793; doi:10.3201/eid2405.171192)
Supplement: Technical Appendix — Sampling locations of dromedary camels, Bangladesh, 2015. [file 17-1192-Techapp-s1.pdf]

Article DOI: https://doi.org/10.3201/eid2405.171192

Middle East Respiratory Syndrome Coronavirus Antibodies in Dromedary Camels, Bangladesh, 2015

Technical Appendix

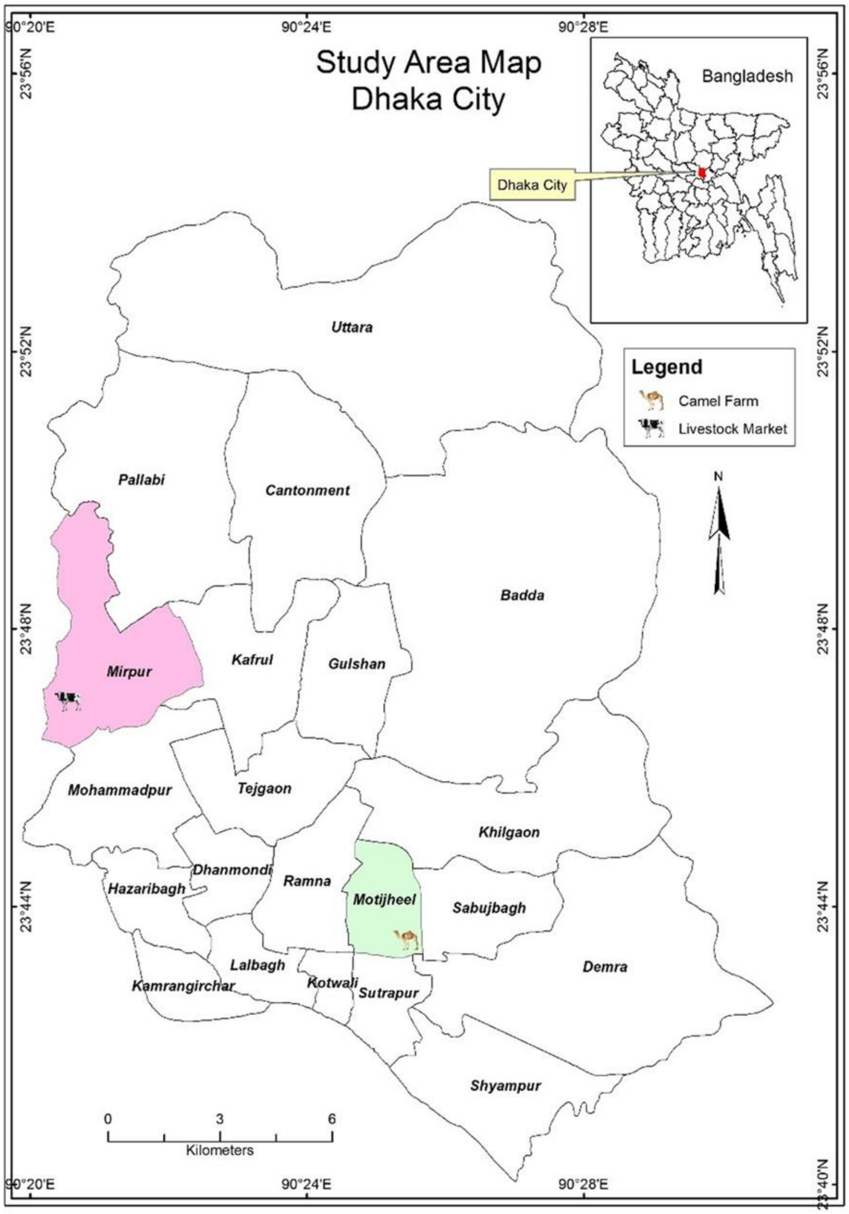

Technical Appendix Figure. Sampling locations of dromedary camels, Bangladesh, 2015.
